# Migrants and imported disease: Trends of admission in an Italian infectious disease ward during the migration crisis of 2015–2017

**DOI:** 10.1186/s12889-020-08886-0

**Published:** 2020-05-20

**Authors:** Filippo Del Puente, Niccolò Riccardi, Lucia Taramasso, Giovanni Sarteschi, Rachele Pincino, Antonio Di Biagio

**Affiliations:** 1 Department of Health Sciences (DiSSal), Infectious Diseases Clinic, IRCCS Ospedale Policlinico San Martino, University of Genoa, Genoa, Italy; 2 Department of Infectious – Tropical Diseases and Microbiology, IRCCS Ospedale Sacro Cuore Don Calabria, Negrar di Valpolicella, Verona, Italy; 3 StopTB Italia Onlus, Milan, Italy; 4 Department of Internal Medicine, Infectious Diseases Unit, Fondazione IRCCS Cà Granda Ospedale Maggiore Policlinico, University of Milan, Milan, Italy

**Keywords:** Migrants, Hospital admission, Italy, LTBI

## Abstract

**Background:**

Since 2014, the migrant population residing in Europe has dramatically increased. Migrants’ unmet health needs represent a barrier to integration and should be promptly addressed, without stigma, in order to favour resettlement.

**Methods:**

All-cause of admissions in the migrant population at the Infectious Disease Clinic of Policlinico San Martino Hospital in Genoa between 2015 and 2017 were analysed. Patients were classified by duration of residence in Italy according to the Recommendation on Statistics of International Migration, cause of hospitalization, and region of origin. All data were evaluated with SPSS Statistics.

**Results:**

Two hundred thirty-five people were admitted, 86 (36.5%) of them residing in Italy for less than 1 year. Except for a significant increase in migrants from Africa, there was no change considering the area of origin, hospitalization reason or by comparing residency in Italy for more or less than 1 year. A considerable number of hospitalizations were related to non-communicable pathologies and latent tuberculosis infection. Residents in Italy for less than 1 year or with active tuberculosis had prolonged hospitalizations, while HIV-infected had shorter hospital stays.

**Conclusions:**

No difference in terms of diagnosis were found between migrants with longer or shorter period of residence in Italy. Adequate outpatient services for the management of communicable diseases could significantly reduce the length of hospitalizations in the migrant population.

## Background

Evaluation of migrants’ health needs is increasingly gaining attention since the beginning of European migrant crisis in 2015 and following the release of the Strategy and Action Plan for Refugee and Migrant Health in 2016 [1], the World Health Assembly resolution A72.25 [2], and the Framework of Priorities and Guiding Principles to Promote the Health of Refugees and Migrants in 2017 [3].

As stated in these programmatic documents, new evidences are required in order to assess needs and provide accurate and reliable information to support efficient and effective approaches for refugee and migrant health, while creating systems of standardized and disaggregated data for all refugees and migrants to support regional and national health policy [1]. In fact, many authors agree that health regulations should be evaluated to be more effective [4], and screening and social policies should be enacted to decrease avoidable hospitalizations and assist migrants with comprehensive follow-up programs [5–7]. To increase medical evidences and to plan fruitful strategies, implementation of migrants medical registries have been proposed [8].

Since national and regional registry designed to focus on migrant health are still unavailable in Italy https://www.simmweb.it/954-scheda-nazionale-accesso-ssn, we decided to gather information regarding migrants’ health needs in the Infectious Disease Unit of the Policlinico San Martino, a large teaching hospital in Genoa, performed during the time period of European migrant crisis (2015–2017).

Genoa, in the North-West of Italy, is considered a hot-spot for the evaluation of migratory flows with more than 54,000 foreign-born citizens living in the metropolitan area in 2017, accounting for the 9.4% of the total population [9] while the Infectious Disease Unit of San Martino Hospital is considered one of the main receptive hub for management of migrant people.

The aim of the present study was to evaluate the cause of hospitalizations of migrants in a large University hospital in Genoa in order to be able to guide future management assessments in this population.

## Methods

We performed a three-year survey (2015–2017) of migrant patients admitted to the Infectious Diseases Clinic of Policlinico San Martino Hospital in Genoa by assessing their primary therapeutic needs (divided according to the diagnosis at discharge), their duration of residence in Italy, the length of hospital stay and region of origin, (i.e., divided in Africa, South America, Eastern Europe or Asia).

Patients were thus divided into residents in Italy for more than one year or “longer residents” (LR) and less than one year or “shorter residents” (SR), according to the Recommendation on Statistics of International Migration with the aim of differentiating the burden of so-called “imported” pathologies and pathologies that arise or reactivate after a prolonged period of residence in Italy [10]. Data regarding nationality and period of residence in Italy were retrieved from the hospital’s internal electronic database. Admissions were identified using the Diagnosis-Related Group (DRG) codes based on the International Classification of Diseases (ICD-10). First empirical evaluation of patients was performed by clinicians in the Emergency Department, people considered to be affected by transmittable disease were then sent to the Infectious disease Unit.

The causes of hospitalization were split into 5 areas (non-communicable diseases, NCDs; active tuberculosis, TB; other communicable diseases, CDs; latent tuberculosis, LTBI, and Human Immunodeficiency Virus, HIV-1). This type of division has been suggested by an initial empirical evaluation of the causes of hospitalization and in order to give adequate prominence to HIV and tuberculosis (TB), which, in terms of stigma and social burden, are of particular importance in the health and anthropological analysis of disease both in migrants and in the resident population [10–13] For clarity reasons, and in order to provide the most accurate real-life profile, we decided to consider not only the leading cause of admission, but all the diagnoses made during hospitalization. For the same reasons, consecutive patients were recorded, without exclusion criteria, except for re-admission of the same patient for the same pathology.

As CDs and NCDs categories include different pathologies, for a better understanding of hospitalization’s reasons, we summarize the frequency and type of diagnosis for both categories in Table 1. In order to provide more complete disclosure of data, the number of patients for each country of origin and region is available in Table 2. Independent samples with continuous data and normal distribution were evaluated by t-test for two independent means, while categorical data were evaluated by the Chi-squared test or Fisher’s exact test. Continuous variables were reported as mean and standard deviation.

**Table 1:** List and number of Communicable diseases (CDs) and non-communicable diseases (nCDs) occurred in foreign-born patients during the 2015–2017 period

CLASSIFICATION	ICD-10	Number (%)
CDs (Communicable diseases)		
Acute respiratory illness (seasonal outbreak)	J22	
Influenza	J09-J10	19 (14)
STD	From A50 to A64	15 (8)
Hepatitis B	B16	14 (7)
Hepatitis A	B15	8 (4)
Severe infection due to *Staphylococcus aureus*	A41.0, A49.0	11 (6)
Acute diarrhoea	A09	10 (5)
FUO	R50	7 (4)
Other		17 (9)
NCDs (Non-communicable diseases)		
*Cardiology*		
Hypertension	I10-I15	18 (9)
Other cardiovascular disease		7 (4)
*Endocrinology*		
Diabetes mellitus	E10-E14	13 (7)
*Haematology And Oncology*		
Haematological and lymphoid tissue neoplasm	From C81 to C96	6 (3)
Solid organ	From C0 to C75	3 (2)
*Pulmonary Disease*		
COPD	J44.9	5 (3)
*Nephrology*		
CKD	N18	5 (3)
AKI	N17	2 (1)
*Gastroenterology*		
IBD	From K50 to K52	4 (2)
Other		2 (1)

*Abbreviations*: *STD* sexually transmitted disease, *FUO* fever of unknown origin, *COPD* chronic-obstructive pulmonary disease, *CKD* chronic kidney disease, *AKI* acute kidney injury

**Table 2:** Macro-area and UN subregion of origin of patients admitted during the 2015–2017 period

Macro-Area	UN Subregion	Number of patients (%)
**Africa**	West Africa	86 (37)
	East Africa	5 (2)
	Central Africa	9 (4)
	North Africa	17 (7)
**Asia**	Southern Asia	6 (3)
	Eastern Asia	2 (1)
	South-eastern Asia	3 (1)
**East Europe**	Ex-Jugoslavia	21 (9)
	Ex-URSS	32 (14
**Latin America**	South America	53 (23)
	Central America	1 (0)

All data have been obtained from information form and electronic medical records and no supplementary patients’ data were asked; informed consent was obtained at the moment of the first visit. All patients signed informed consent at admission to our Institution for the use of clinical data in the case of retrospective observational study, according to the Regione Liguria ethical committee for data acquisition at the Policlinico San Martino Hospital. All data were anonymized and evaluated with SPSS Statistics. Every patient signed the informed consent for the scientific use of clinical data.

## Results

During the observation period, a total of 6894 admissions were registered, 235 for foreign-born patients (3.04%), of these, 86 (36.5%) were SR (63 patients from Africa; 9 from Eastern Europe; 8 from South America and 4 from Asia). Compared to SR, LR patients were significantly younger (41.12 ± 14.10 and 27.32 ± 10.06, *p* < 0.001).

Considering the overall situation, a significant increase in hospitalizations of patients from Africa was observed both in absolute terms (*p* < 0.001), and by comparing it to other regions, representing, in 2015, 40.98% of total hospitalizations for foreign-born patients; 51.09% in 2016, and 54.88% in 2017. We also registered a significant but steady proportion of patients coming from South America and Eastern Europe, while a limited number of patients from Asia were admitted (see Table 3).

**Table 3:** Number of admissions divided per macro-area and year

	2015	2016	2017	Total (%)	
**Africa**	25 (41)^a^	47 (51)^a^	45 (55)^a^	117 (50)^b^	p < 0.001
**Asia**	4 (6)^a^	2 (2)^a^	5 (6)^a^	11 (5)^b^	*p* = 0.427
**Eastern Europe**	15 (25)^a^	25 (27)^a^	13 (16)^a^	53 (23)^b^	*p* = 0.278
**Latin America**	17 (28)^a^	18 (20)^a^	19 (23)^a^	54 (23)^b^	*p* = 0.355
**Total**	61 (26)^b^	92 (39)^b^	82 (35)^b^	235	

Highlighted as ^a^ are percentages referring to the total for the single year, as ^b^ the percentages referring to the total of the patients

By analysing the causes of hospitalization, we observed no significant change during the observation period, neither in general terms nor in terms of a single region (see Table 4). Interestingly, causes of hospitalization for SR and LR patients did not significantly differ (see Table 5). An elevated proportion of admissions (14.08%) were registered as due to LTBI, while 18.68% of admissions were due to NCDs. Regarding HIV, we observed that 52 patients out of 235 were seropositive, with a prevalence of 22.13%.

**Table 4:** Number of admissions divided per macro-area and diagnosis. In brackets are the percentages referring to the total of the macro-area

	Africa	Asia	East Europe	Latin America
**HIV**	17 (12)	1 (7)	11 (11)	23 (24)
**CDs**	59 (12)	9 (64)	33 (34)	26 (28)
**NCDs**	32 (22)	3 (2)	12 (12)	18 (19)
**TB latent**	7 (5)	1 (7)	28 (29)	13 (14)
**TB active**	28 (20)	0	13 (13)	14 (15)
**Total**	143	14	97	94

**Table 5:** Difference in terms of reason for admission between SR and LR. In brackets the percentages referring to the total population

	HIV	CDs	NCDs	TB latent	TB active	Total
**SR**	15 (13)	39 (33)	20 (17)	14 (12)	29 (25)	117
**LR**	37 (16)	88 (38)	45 (19)	35 (15)	26 (11)	231
**Total**	52	127	65	49	55	348

*Abbreviations*: *SR* shorter residents, *LR* longer residents

By analysing the duration of hospitalization, we observed longer period for patients admitted for active TB compared to patients admitted for other reasons (28.12 ± 11.64 vs. 16.72 ± 8.07 days, *p* < 0.0001) and in HIV-1 uninfected patients compared to HIV-1 infected patients (23.76 ± 11.08 vs. 18.67 ± 9.38 days, *p* = 0.004) and in patients without CDs compared to patients with CDs (23.03 ± 10.85 vs. 16.38 ± 7.66 days, *p* < 0.0001). No significant differences were found in lengths of hospital stay between patients coming from different regions, while LR were found to have significantly shorter periods of hospitalization compared to SR (17.01 ± 8.63 vs. 23.03 ± 10.52 days, *p* < 0.0001).

Considered the elevated proportion of patients coming from Africa, the analysis performed, in terms of causes of admissions and length of hospitalization, between African and non-African patients did not reveal any significant difference.

## Discussion

This real-life observational and retrospective study focused on assessing hospital care needs in the migrant population in a large teaching hospital serving a metropolitan area of Northern Italy. An increasing number in admissions of foreign patients was observed, mostly due to the growing number of admissions for patients arrived for less than a year, mostly persons of African origin. Interestingly, the incidence of the various types of pathologies (CDs, NCDs, TB, LTBI, and HIV) did not change between the cohort of SR and LR patients. This finding is in agreement with the trend registered in several other Countries [1, 12–14] and contribute to the suggestion that migrants are not a potential epidemiological danger per se for the emergence or re-emergence of communicable diseases, such as TB or HIV, in developed countries.

The longer duration of hospitalizations in SR suggests that these patients are an “hard-to-diagnose” and “hard-to-treat” subgroup of people, possibly for lack of targeted policies. In particular, the absence a social policy designed for these patients often translate in longer period of hospitalization, higher costs, risk of nosocomial complications [15] and, from a more psychological perspective, a sense of neglect for both patients and caregivers.

Along with these considerations, it should be noted that patients discharged with the diagnosis of active TB are commonly treated for a longer period in hospital than people without active TB. This finding can be only partially explained by the necessity of physical isolation as the mean duration of hospitalization in this group is 28 days, thus double the time usually needed to remove patients from respiratory isolation. On the contrary, HIV-infected patients had a significantly shorter duration of hospitalization. These findings are possibly explained by the presence, in our country and particularly in the centre of Genoa, of a functional outpatient service for HIV infected patients that possibly allows to discharge HIV-infected patients with a scheduled follow-up safely. Due to the absence of a significant epidemiology in terms of TB, this service has not been adapted so far for people affected by this illness, making discharge more difficult especially in patients without the appropriate social security or the cultural means to follow the appropriate treatment schedule.

LTBI, due to the high prevalence of active tuberculosis in the countries of origin, is a prominent finding during hospitalization screening. This finding is a particularly worrisome moment, as LTBI should need targeted assessment, therapy and follow-up at the points of entry and after re-allocation and should not warrant hospitalization [16, 17]. A similar problem was observed for patients admitted for NCDs due to the misconception of migration as a source for infectious diseases [18].

In order to analyse the epidemiological picture in a comprehensive way, it is worthy of mention that the average age of patients from the various regions did not vary significantly, although showing a trend for younger ages for patients from Africa and Asia, with this observation probably influenced by the high proportion of African patients considered as SR (see Table 6).

**Table 6:** Macro-area of origin, number of admissions per year and median age at admission

	Sex (%)	Number of total admission	Median age at admission (range)
**Africa**	92 M (79), 25 F (21)	117	27 (17–62)
**Asia**	6 M (54), 5 F (45)	11	32 (25–56)
**East Europa**	31 M (58), 22 F (42)	53	36 (28–70)
**Latin America**	27 M (50), 27 F (50)	54	35 (29–56)
**Total**	156 M (66), 79 F (34)	235	34 (17–70)

*Abbreviations: M* male, *F* female

The increasing rate of hospitalization of people from Africa is probably attributable to the social and geographical reasons of the migrant crisis between 2015 and 2017, as most people coming to Italy is of African origin.

Limits of the present study are its retrospective nature and the absence of follow up and outcome data; the admission rate was not possible to retrieve due to the hospital electronic database that does not cover the Emergency Department and the outpatient clinics. In order to differentiate patients, a one-year cut-off value was considered [18]. Also, this survey, carried out in a hospital setting, did not consider patients managed by outpatient and extra-hospital services.

## Conclusion

With these limitations, our work shows that in the face of an increase in migratory flows, there has not been a relative increase in communicable diseases that required hospitalization, even if there is a high number of admissions in absolute terms. Seroprevalence of HIV among migrants was high, even if we cannot rule out the hypothesis of more cases gone undiagnosed in migrants who did not seek hospital care. We also report a seemingly counterintuitive high proportion of LTBI diagnosis performed in-hospital, as this diagnosis and its management should be theoretically performed by outpatient services.

Careful screening at entry points coupled with patients’ allocation to the correct ward may be essential to reduce inappropriate hospitalizations and hospitalization length.

Finally, improving outpatient-care centres is highly suggested in order to reduce the rate of unnecessary hospitalization and possibly guarantee better management, with dedicated follow-up, of infectious diseases in the migrant population.

## Data Availability

The datasets used and/or analysed during the current study are available from the corresponding author on reasonable request.

## Abbreviations

CDs: Communicable diseases
DRG: Diagnosis-Related Group
HIV: Human Immunodeficiency Virus
ICD-10: International Classification of Diseases
LTBI: Latent Tuberculosis Infection
nodes: Non-Communicable Diseases
TB: Tuberculosis
